# Animal Welfare and Farmers' Satisfaction in Small-Scale Dairy Farms in the Eastern Alps: A “One Welfare” Approach

**DOI:** 10.3389/fvets.2021.741497

**Published:** 2021-11-10

**Authors:** Chiara Spigarelli, Marco Berton, Mirco Corazzin, Luigi Gallo, Sabine Pinterits, Maurizio Ramanzin, Wolfgang Ressi, Enrico Sturaro, Anna Zuliani, Stefano Bovolenta

**Affiliations:** ^1^Department of Agricultural, Food, Environmental and Animal Sciences, University of Udine, Udine, Italy; ^2^Department of Agronomy, Food, Natural Resources, Animals and Environment, University of Padova, Padova, Italy; ^3^Umweltbüro, Klagenfurt, Austria

**Keywords:** mountain livestock farming, dairy cattle, animal welfare, farm type, One Welfare

## Abstract

The multidimensional concept of animal welfare includes physical health, good emotional state, and appropriate behavior of the animals. The most recent methods for its assessment are inspired by the Welfare Quality, a project compiling animal-, resource-, and management-based measures. Recently, animal welfare assessment has also considered the human factor in a so-called “One Welfare” approach. The One Welfare framework highlights the interconnections between animal welfare, human well-being, and the environment. The concept seems to fit particularly well to mountain areas where the relationship between human, animals, and the environment is stronger. In such disadvantaged areas, farmers' well-being plays a key role in maintaining livestock farming profitably and sustainably. This study aims to investigate the relationship between farmers' satisfaction, animal welfare outcomes, and overall farm performance in 69 small-scale dairy farms in the Eastern Alps. Animal welfare assessment consisted of animal-based measures and was performed using the methodology proposed by the European Food Safety Authority for this type of farm. Moreover, the farmers were interviewed to retrieve data on farm characteristics and on their level of satisfaction toward workload, land organization, relationship with the agricultural/non-agricultural community, and the future of local agriculture. The results show that good animal welfare can be obtained in a mountain farming system. Clinical indicators show a low prevalence of diseases and of very lean cows as opposed to integument alterations. The workload is not perceived as a problem in traditional farms (i.e., tie-stall and with no participation in quality-certification schemes). Animal welfare is higher in those farms where farmers have a positive engagement with both the agricultural and non-agricultural community and where farmers are satisfied with their land organization. A One Welfare approach could be applied on a larger scale to fully understand the links between animal and human well-being in mountain areas.

## Introduction

Farm animal welfare is an ever-evolving, multidimensional concept—not easy to define and evaluate (1). However, public awareness about this issue has progressively increased in recent decades (2). Animal welfare as a “formal discipline” started with the publication of the “five freedoms” proposed by the (3). Since then, much progress has been achieved. Broom (4) links animal welfare to the attempts to cope with the environment, and Webster (5) introduces also the concept of physical and mental health. Furthermore, Fraser (6) focuses on the possibility that animals suffer from the mere fact of being kept under “unnatural” conditions. A very large amount of research has been carried out about animal welfare, particularly focusing on the development of welfare-assessment methods in different environments (7). Many of these research findings contributed to the assessment protocols produced by the Welfare Quality project (8), the largest study carried out in the EU to develop scientifically based measures for farm animal welfare and to convert these into accessible and understandable information (9). The Welfare Quality assessment protocol combines animal-, resource-, and management-based measures to distinguish four principles—good feeding, good housing, good health, and appropriate behavior—and identify an overall degree of welfare.

Although the Welfare Quality assessment protocol is the basis for the most recent assessment methods for cattle used in Europe, it is mainly suited for indoor and intensive livestock farming systems, and the proposed measures are often very difficult to collect in extensive and small-scale farming systems. For this reason and to address the lack of information regarding animal welfare in these contexts, the European Food Safety Authority (EFSA) published a scientific opinion on the feasibility of current welfare assessment methods in so-called “non-conventional small-scale dairy farming settings” (10). One of the outcomes of the scientific opinion was a protocol that relies mainly on animal-based measures (ABM) for the evaluation of well-being and not on the diversified housing and management strategies that characterize these livestock systems. The approach has also been used by the World Organization of Animal Health (11) and by the International Standardization Organization (12).

Dairy mountain farms represent one example of small-scale livestock farming settings (13), where animal welfare assessment can be carried out on-farm (14, 15) and also at pasture (16, 17). In mountain regions, livestock farming has traditionally been of great importance for the vitality of rural economies (18, 19). Livestock systems help to shape mountain landscapes, providing ecosystem services (20). In the Alpine region, mountain farming has profoundly changed in recent decades as a consequence of dissimilarities in local policies and socioeconomic conditions. As reported by Battaglini et al. (21), farm abandonment in unfavorable locations vs. intensification of farm operations in favorable sites has often weakened the link between livestock, farmers, and grasslands resources. Farmers' knowledge, attitudes, skills, and familiarity with livestock are important, but broader aspects, such as job motivation and satisfaction, working conditions and regulations, and relationships with coworkers and the wider community, play a key role in viable farming (22). Farming is considered a stressful occupation because of its workload, financial challenges, overwhelming administrative procedures, and imposition of new regulations (23, 24). In addition, dealing with unfavorable weather (25) as well as lack of understanding from the non-agricultural community are also among the stressors reported (24). Some research also highlights the importance of loneliness and geographical isolation as sources of stress because farmers have even fewer opportunities and means to control external factors (23, 26).

It is suggested that farmers' job satisfaction and motivation may have a high influence on animal welfare status (27). A recent Canadian study reports the association between animal welfare outcomes and productivity and profitability of farming (28). The recognition of the link between animal and human welfare has paved the way to the development of the “One Welfare” approach (29) as an interdisciplinary concept of welfare. This approach helps to recognize the connections between animal welfare and human well-being more effectively in various areas of human society, including environmental science and sustainability (30). For Hemsworth and Coleman (31), the quality of stockmanship contributes to the human–animal relationship, animal welfare, and productivity. Attitudes can affect the way farmers treat their animals, the environment they provide for the animals, and even their own job satisfaction through the feedback received from the animals (32).

With the proposed “One Welfare” framework in mind, this study investigates the relationship between dairy cattle animal welfare outcomes, farmers' satisfaction, and the overall farm performance in small-scale farms in the Eastern Alps.

## Materials and Methods

### Farm Characteristics

The study was conducted in 69 mountain dairy farms in the Eastern Alps. Farms involved were members of the Breeders Association, took part in the milk recording program, and can be considered as small-scale farms because of the maximum number of animals reared (75 dairy cows) and the family workforce. Farms were visited twice during 2018 for data collection.

In a first visit, the farmer was interviewed by a researcher to retrieve data on farm characteristics and farm records. Data collection focused on the information regarding farmers' age and the following farm descriptors: presence of quality certification schemes [Regulation (EU) No 1151/2012 and/or Regulation (EC) No 834/2007], type of housing systems (loose housing or tie-stall), farm production in terms of milk yield (i.e., kg FPCM -*fat and protein corrected milk (3.3% protein and 4.0% fat*-, milk price (€/kg excluding VAT), income from milk production on total income (percentage of total), stocking rate (LU/ha UAA—*Livestock Unit/ha Utilized Agricultural Area*-), and forage and feed self-sufficiency (percentage).

### Animal Welfare Assessment

During the second visit, animal welfare assessment was performed on 1,584 dairy cows by one observer trained in animal welfare science during the fall/winter seasons when all animals were housed indoors. The assessment protocol for ABM followed the aforementioned methodology proposed by EFSA for small-scale dairy farms (10). The EFSA protocol differs from the Welfare Quality assessment protocol in regards to some measures: record of coughing episodes was removed from the protocol as the EFSA panel considered the evaluation of this measure too time-consuming. Instead, two additional measures were added: longevity (expressed as the percentage of cows in the fourth lactation or higher) and claw condition (classified as “good condition” or “overgrown”). The measure regarding ocular discharge was redefined by adding a new category (i.e., distinguishing between serous and purulent ocular discharge), and teats were considered separately from the rest of the udder when scoring for soiling.

All ABMs were divided into ABMs observed (ABMo) and ABMs recorded (ABMr) from milk records. The former included body condition score (BCS), soiling, integument alterations (hairless patches, lesions, swellings, and claw overgrowth), and clinical conditions (lameness/severe lameness, ocular discharge, nasal discharge, vulvar discharge, hampered respiration, and diarrhea). In terms of behavioral measures, Qualitative Behavior Assessment (QBA) and Avoidance Distance at the Feeding place (ADF) were collected. For QBA, 20 behavioral descriptors were weighted and aggregated into a QBA index ranging from 0 to 100 by computing the weighted sum as described in (8). The ADF was measured by assessing the flee distance in centimeters. Only the number of animals that were touched was collected. The ABMr aimed at retrieving information on longevity, incidence of downer cows, dystocia, sudden deaths, or emergency slaughter/euthanasia (i.e., “mortality”) and milk somatic cell count (SCC > 400,000 cells/mL) from milk records during a 12-month-period. Animal-level measurements were collected according to (8) guidelines for sample size calculation.

### Farmers' Satisfaction

To describe the level of farmers' satisfaction, the participants were also asked to answer five questions based on a Likert scale (1 = extremely unsatisfied to 5 = extremely satisfied) (33). The questions were taken from the protocol proposed by (34) and concerned the perception about the amount of work (WL; question: *Are you satisfied with the work load?*), the land organization (LO; question: *Are you satisfied with the land organization of your farm?*), the relationship with the non-agricultural community (RNAC; question: *Are you satisfied with the relationship with the municipality and the population?*) or agricultural community (RAC; question: *Are you satisfied with the relationship with local economic operators and other farmers?*), and finally, the future of local agriculture (FA; question: *Are you satisfied with how you see the future of local agriculture?*).

### Data Analysis

Data were analyzed in R (3.4.0 version, R core team, 2017). Prevalence was computed on all ABM variables collected to identify critical or achievable levels applicable to small-scale mountain dairy farms (35). Variables were classified according to their position on the distribution. A value between 1 and 4 was assigned to each quartile, where 4 represents the highest level of welfare. Quartiles were scored in decreasing order, e.g., the first quartile (lowest ABM prevalence) was scored with the highest welfare value (6) and the last quartile (highest ABM prevalence) with the lowest welfare value (2) with the exceptions of ADF, QBA, and longevity, which quartiles were scored in increasing order. The overall animal welfare index was the result of the sum of all assigned values. The differences among the response categories related to farmers' satisfaction were assessed with an exact multinomial test, and *post hoc* exact binomial tests with Holm correction for pairwise comparisons were also performed using the RVAideMemoire package (36).

To explore the relationship between farmer satisfaction, animal welfare index, and the variables describing farms characteristics, a principal component analysis (PCA) was used. For this purpose, the levels of farmers satisfaction based on a 5-point Likert scale were grouped into three categories: unsatisfied (WLl, LOl, FAl, RNACl, RACl, Likert scale 1 and 2), neutral (WLm, LOm, FAm, RNACm, RACm, Likert scale 3), and satisfied (WLh, LOh, FAh, RNACh, RACh, Likert scale 4 and 5). Farm characteristics included in the analysis were age of farmer, presence of quality certification schemes, type of housing systems, milk yield, milk price, dairy income, stocking rate, feed self-sufficiency, and forage self-sufficiency. Only the components with eigenvalues >1 were retained in the analysis. The PCA was carried out with the PCAmixdata package (37) that allowed considering both continuous and categorical variables, and the PCArot function was used to improve the clarity of the data interpretation (38).

## Results

### Farm Performance

Descriptive statistics relating to the farms identified for the study are shown in Table 1.

**Table 1:** Characteristics of dairy farms involved (*n* = 69).

**Explanatory variable**	**Min**	**25 Perc**	**Median**	**75 Perc**	**Max**
Age of farmers (year)	21	35	45	55	73
Lactating cows/farm (n.)	4	13	22	30	70
Farm elevation (m a.s.l.)	280	604	776	969	1,375
Milk production (kg FPCM^1^)	3,758	6,483	7,468	8,699	10,336
Milk price (€/kg)	0.32	0.40	0.46	0.56	0.82
Dairy income (% of total)	20	50	80	100	100
Forage self-sufficiency (%)	45.0	65.8	69.6	87.9	100
Feed self-sufficiency (%)	33.0	55.3	61.0	79.6	95.2
Stocking rate (LU/ha UAA)^2^	0.50	0.85	1.21	1.72	4.14

1 *Fat- and protein-corrected milk (3.3% protein and 4.0% fat)*;

2 *Livestock Unit / Utilized Agricultural Area*.

The median farmer's age was 45, and 66% of them were more than 40 years old. All farms had fewer than 75 cows, and they were all located in mountain areas, the majority at below than 1,000 m a.s.l. Milk yield ranged between 3,758 and 10,336 kg of FPCM. Sixty percent of the farms had Simmental cows, followed by Holstein in 26% of the farms, and crossbreeds in the remainder. The proportion of farms keeping multiple breeds was 1:3. Milk price ranged between 0.32 and 0.82 euro/kg, and for 56% of the farmers, their income derived exclusively or almost exclusively (≥80% of the total income) from farming. The majority of farms were forage self-sufficient, and 80% of the farms had a livestock density of <2 a LU/ha UAA. Most farms had feed self-sufficiency levels >50%. The median prevalence of self-sufficiency was 69 with 33 farms being higher than 90% and only 10 farms relying mostly on external inputs.

Considering the categorical variables collected (not reported in tables), 43 farms used loose stalls (62% of the total), and only 26 farms used the tie-stall system. Seventy-four percent of farmers did not participate in any product quality scheme, only one farm was associated with the Traditional Specialty Guaranteed (TSG) quality scheme for hay milk production, and 15 farms followed organic farming practices.

### Animal Welfare Assessment

Summary statistics for ABMo and ABMr are given in Table 2.

**Table 2:** Prevalence of ABMs observed or retrieved from farm records in 69 mountain dairy farms.

**ABMs**	**Min**	**25 Perc**	**Median**	**75 Perc**	**Max**
Very lean	0	0	3	7	69
Dirty legs	0	6	18	50	88
Dirty teats	0	0	6	14	75
Hairless legs	0	17	31	50	94
Hairless body	0	6	14	32	71
Lesions and swellings	0	3	9	17	58
Nasal discharges	0	0	0	0	10
Ocular discharges	0	0	0	3	38
Vulvar discharges	0	0	0	0	8
Hampered respiration	0	0	0	0	6
Diarrhea	0	0	0	0	44
Severe lameness	0	0	0	7	53
ADF^1^ = 0	10	69	81	92	100
QBA^2^	0	28	48	67	89
Longevity	0	20	29	38	78
Dystocia	0	0	0	4	18
Downer	0	0	0	6	22
SCC^3^ > 400.000 cells/mL	0	4	8	12	29
Mortality	0	0	0	4	13

1 *Avoidance Distance at Feeding place*;

2 *Qualitative Behavior Assessment*;

3 *Somatic Cell Count*.

Concerning the lean cow assessment, the median prevalence of lean cows was 3%; the third quartile had a prevalence from 7% up to 69%. Dirtiness indicators had a median value of 18 and 6% for legs and teats, respectively, and the median prevalence values of cows with high SCC count and severe lameness were also low (8 and 0%). Median values were 31% of animals with hairless legs, 14% with hairless body, and 9% with lesions and swellings with maximum values that reached 94, 71, and 58% of affected animals, respectively. Few cases of nasal, vulvar, and ocular discharges, hampered respiration, and diarrhea were observed. The median prevalence of discharges was 0% except for the upper 25% of the farms with values from 3 to 38% of animals with an ocular discharge. Regarding ADF assessment, the results showed a median prevalence of 81% of animals touched; the QBA index had a median of 49 (range: 0–100). Low prevalence was also found in dystocia, downer cows, and mortality as compared with longevity with 29% of animals above the third lactation.

### Farmers' Satisfaction

About LO, nearly 40% of farmers were extremely satisfied, a much greater percentage than that observed for extremely and slightly unsatisfied (*P* < 0.05; Table 3). No farmers were extremely unsatisfied with RNAC, and the percentage of extremely satisfied farmers was higher than the percentage of slightly unsatisfied farmers (*P* < 0.05). Overall, farmers were also particularly satisfied with RAC; indeed, the percentage of respondents who were slightly or extremely satisfied was significantly higher than the percentage of respondents who were slightly or extremely unsatisfied (*P* < 0.05; Table 3).

**Table 3:** Farmers' satisfaction (frequency, %) in relation to different issues.

	**Issues**				
	**WL**	**LO**	**FA**	**RNAC**	**RAC**
Extremely unsatisfied	15.9	10.1^a^	11.6	0.0^a^	4.4^a^
Slightly unsatisfied	20.3	8.7^a^	21.7	13.0^b^	4.4^a^
Neutral	30.4	14.5^ab^	29.0	21.7^b^^c^	13.0^ab^
Slightly satisfied	15.9	29.0^ab^	23.2	26.1^b^^c^	30.4^b^^c^
Extremely satisfied	17.4	37.7^b^	14.5	39.1^c^	47.8^c^
*P*-value	0.330	<0.001	0.148	<0.001	<0.001

a,b *Within columns, values with different superscript letters differ at P < 0·05; WL, amount of work; LO, land organization; RNAC, relationship with non-agricultural community; RAC, relationship with agricultural community; FA, future of local agriculture*.

**Figure 1:**
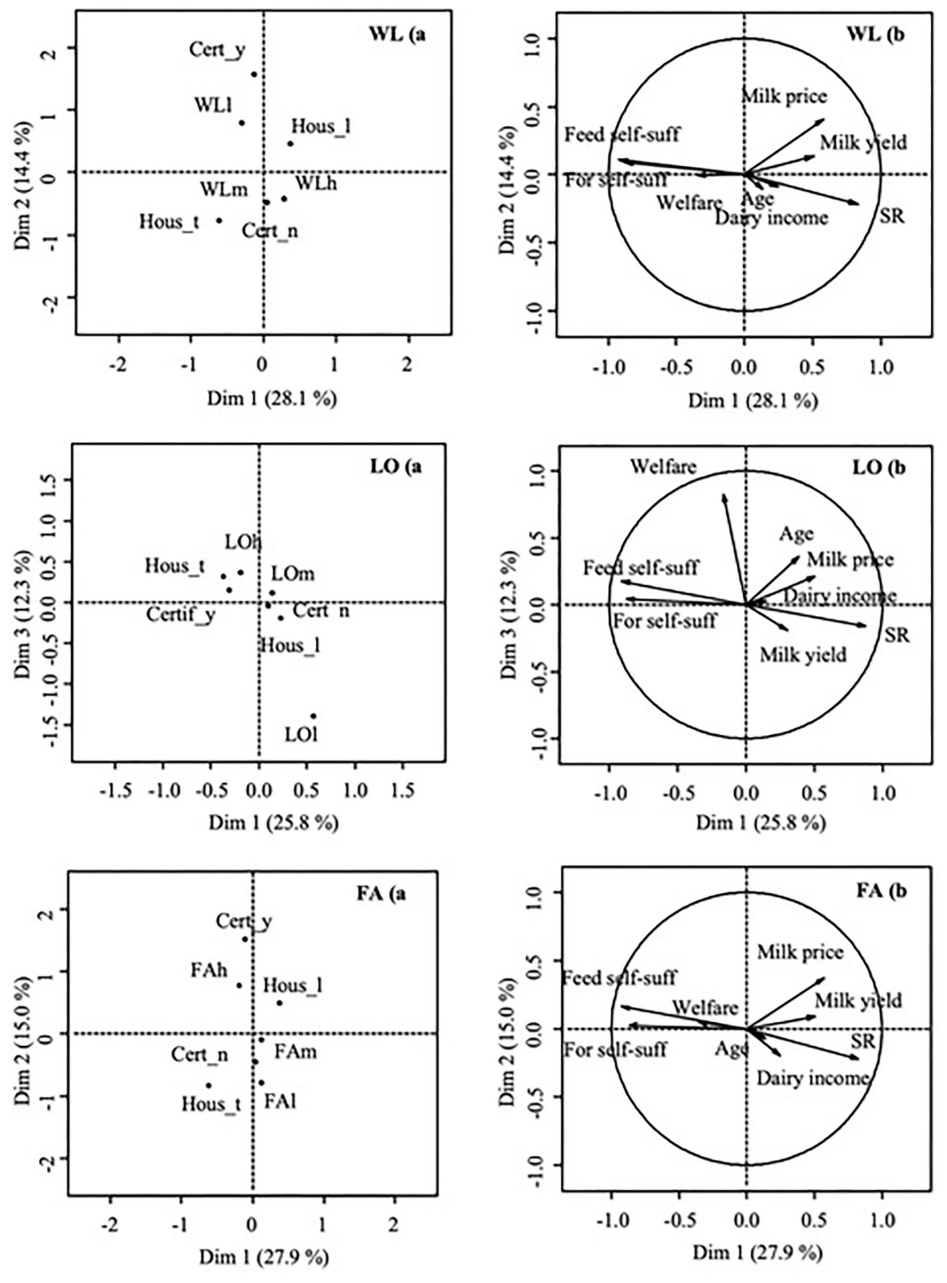
Results of the PCA analysis for the variable related to farmers' satisfaction (h: satisfied, m: neutral, l: unsatisfied) related to WL (amount of workload), LO (land organization), and FA (future of local agriculture); **(A)**: component map with factor scores of categorical variables; **(B)**: component map with factor scores of continuous numerical variables. Cert_y: farms with a certification; Cert_n: farms without certification; Hous_t: tie-stall barn; Hous_l: loose housing farms; Feed self-suff: feed self-sufficiency of farms (%); For self-suff: forage self-sufficiency of farms (%); Welfare: index of animals welfare (points); Age: age of the farmer (years); Dairy income: farm incomes related to the milk production (%); SR: stocking rate (LU/ha UAA); Milk Yield: milk produced per animal per year (kg FPCM); Milk price: market price of the milk (€).

**Figure 2:**
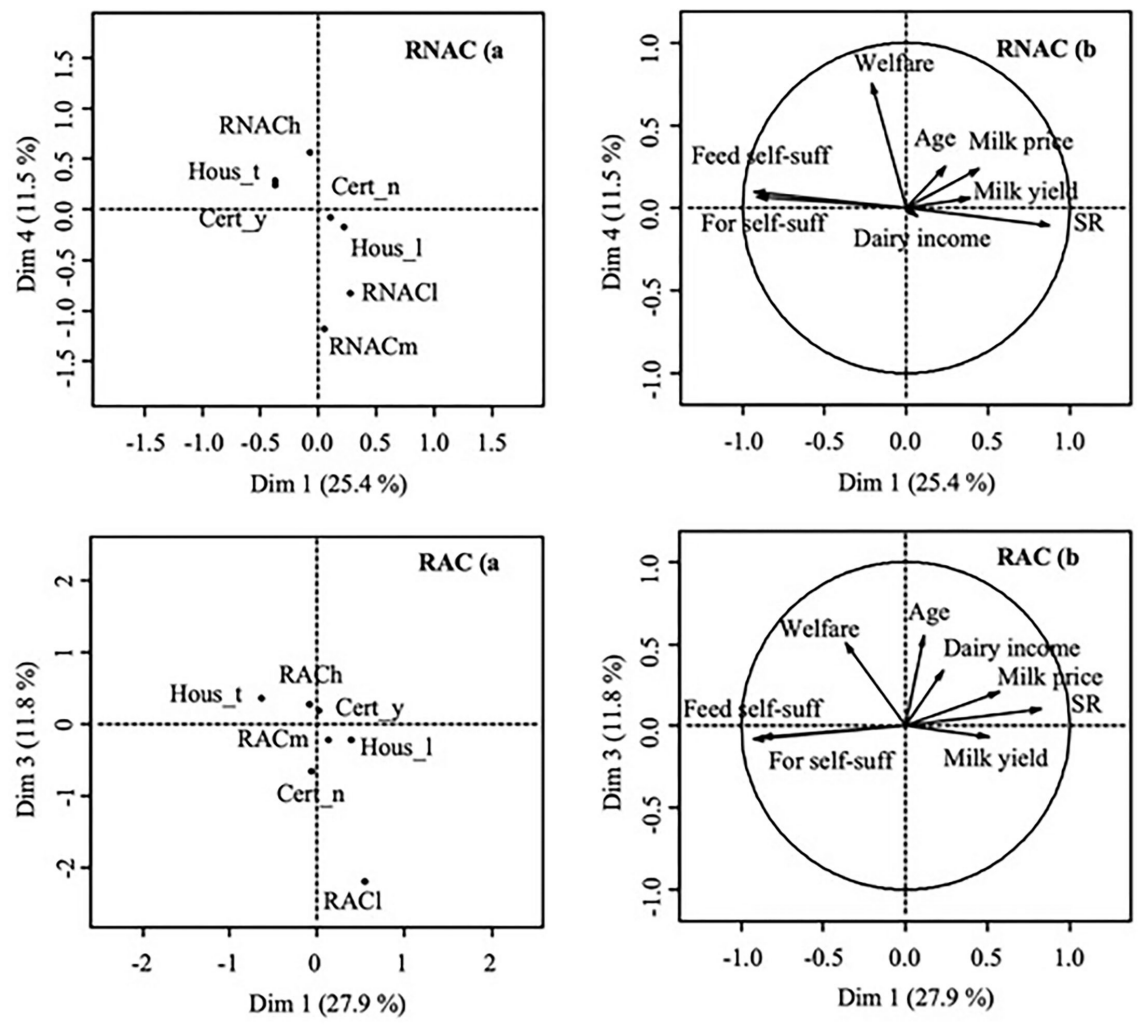
Results of the PCA analysis for the variable related to farmers' satisfaction (h: satisfied, m: neutral, l: unsatisfied) related to the relationship with the nonagricultural (RNAC) and agricultural community (RAC); **(A)**: component map with factor scores of categorical variables; **(B)**: component map with factor scores of continuous numerical variables. Cert_y: farms with a certification; Cert_n: farms without certification; Hous_t: tie-stall barn; Hous_l: loose housing farms; Feed self-suff: feed self-sufficiency of farms (%); For self-suff: forage self-sufficiency of farms (%); Welfare: index of animals welfare (points); Age: age of the farmer (years); Dairy income: farm incomes related to the milk production (%); SR: stocking rate (LU/ha UAA); Milk Yield: milk produced per animal per year (kg FPCM); Milk price: market price of the milk (€).

As shown in Figures 1, 2, the principal components showed the highest correlations with farmers' satisfaction and were able to discriminate between satisfied and unsatisfied farmers, which explained a limited percentage of the total variance.

More specifically, WL is mainly linked to the second component (14% of the variance explained; Figure 1) with a squared loading (SL) of 0.36. WLh is discriminated by WLl mainly by quality certification scheme (SL: 0.74) and housing system (SL: 0.36). In particular, WLh is associated with tie-stall not-certificated farms. LO is mainly linked to the third component (12% of the variance explained, Figure 1) with an SL of 0.46. LOh is discriminated by LOl on the third component mainly by animal welfare (SL: 0.68) to which it is positively associated. FA is mainly linked to the second component (15% of the variance explained, Figure 1) with a SL of 0.43. FAh is discriminated by FAm and FAl on the second component mainly by farm certification (SL: 0.69) and housing system (SL: 0.41).

RNAC is mainly linked to the fourth component (12% of the variance explained, Figure 2) with an SL of 0.60. RNACh is discriminated by RNACl and RNACm on the fourth component mainly by animal welfare (SL: 0.57) with which it was positively related. RAC is mainly linked to the third component (12% of the variance explained, Figure 2) with an SL of 0.48. RACh is discriminated by RACm and RACl on the third component mainly by age of the farmer (SL: 0.30) and animal welfare (SL: 0.25) with which it was positively related.

## Discussion

### Farm Performance

Data on farm characteristics were highly heterogeneus among the sample. In particular, the different breeds reared could contribute to explaining the wide range of production levels, and the socioeconomic and policy conditions of the different areas could explain the great variability of milk prices. Even (39), in a study conducted in the Italian Eastern Alps, highlighted an extreme heterogeneity of farms and dairy supply chains.

Regarding farmer's age, most of them were more than 40 years old. For (40) farmers older than 40 years old have lower willingness to take on farm risks as compared with younger farmers and age potentially influences perceived values, farming objectives, past management decisions, and future intentions.

The low stocking rate observed in the investigated farms could have led to high values of feed and forage self-sufficiency. Berton et al. (41) reports that these variables were negatively correlated in mountain farms, and Penati et al. (42) argues that feed self-sufficiency can be improved also by increasing the use of highland pastures during summer. Maintaining the traditional mountain forage-based systems and improving forage self-sufficiency have positive effects on landscape quality as well as on the conservative functions of managed areas (43, 44). Several studies (45, 46) show that the increase in forage self-sufficiency at the farm level is often linked to a decrease in the overall biodiversity of grasslands.

Despite it being well-known that quality labels, including the optional quality term “mountain product,” are very useful for increasing the added value of mountain products in large-scale food distribution (47), participation in the European quality schemes by the farms involved in the study is very limited. Farmers involved in this study successfully promote their products mainly in local markets as high-quality niche products, directly providing information on production methods, the mountain environment, and product healthiness. In this way, it is possible to avoid the costs related to the quality scheme application.

### Animal Welfare Assessment

In the past, many assessment methods have been used to evaluate farm animal welfare using direct or indirect indicators or a mix. For instance, the Animal Needs Index (ANI) (German Tiergerechtheitsindex—TGI) was developed by Bartussek in Austria (48, 49), where not only housing condition was considered, but also selected aspects of the animal's environment and farm management were used in the indexing method. Bartussek promoted stockmanship as an indicator of the human influence on animal welfare, and the ANI-system influenced how public discussion strongly improved the broad acceptance of the index. Direct measures on animals were mainly used in this study as they were considered more suitable for animal welfare evaluation on small-scale farms as suggested by EFSA.

The results showed a low prevalence of very lean cows on most farms despite higher prevalence values observed by (50, 51) in transhumant systems in the Eastern Alps. Dirtiness indicators suggest that cows were clean in contrast with the findings reported by (52) in similar conditions. Leg and, most importantly, teat cleanliness play a key role in preventing health and production issues, such as mastitis, high SCC in milk, and lameness (53, 54). On the other hand, the prevalence of integument alterations (hairless patches, lesions, or swelling) on both legs and body was lower than that reported by (55) in tie-stall housing, but higher compared with that observed at pasture by (52). This may reflect a higher frequency of collisions against housing structures during lying down in a tie-stall barn. Similar results were obtained in several studies in both indoor systems with access to pasture (35, 56) and outdoor farming systems (52). Human–animal relationships and the emotional state of the animals were in line with previous findings describing a better status in small-scale and tie-stall systems compared to intensive and loose-housing farms (13, 57). The prevalence of ABMs retrieved from milk or farm records (longevity, dystocia, downer cows, and mortality) may be linked to the clinical findings. In fact, a low prevalence of severely lame cows, mastitis, and problems at calving might have contributed to higher longevity (median was at 29% of animals above the third lactation).

### Farmers' Satisfaction

Overall, farmers showed highly variable levels of satisfaction concerning WL and FA with a similar percentage of respondents being satisfied and unsatisfied. Conversely, for LO, RNAC, and RAC, farmers who were satisfied outweighed those who were unsatisfied.

To visualize and explore the relationships between farmers' satisfaction and farm characteristics and animal welfare, a PCA was performed. The variables related to farm characteristics and animal welfare were moderately associated with these principal components. In other words, in general, the possibility to explain the differences in the categories of satisfaction of the respondents with the variables considered is often limited. Flores and Sarandon (58) explain that farmers' satisfaction is strongly contributing to the overall sustainability of livestock farming. Coughenour and Swanson (59) find a connection between satisfaction and farmers' perceptions of the economic and non-economic rewards of farming. However, Herrera et al. (60) observed that the joint effect of farm-level variables, such as working hours, age of assets, and social engagements, was able to explain <20% of the variance of the farmers' satisfaction. The satisfaction about work load seems complex. It may be expected that the highest satisfaction should be associated with more free time for the farmer. Reissig et al. (61) show a higher workload in organic than conventional farms. However, the association between WLh and tie-stall farms did not confirm this hypothesis. Indeed, Poulopoulou et al. (62) report, in mountain farms, a total working time requirement of 177 and of 113 manpower hours per cow per year in tie-stall and loose housing systems, respectively. On the other hand, tie-stall farms are smaller in size and, thus, might be perceived as easier to manage. In addition, tie-stall farms were mainly owned by older farmers, who might be more used to the traditional hardworking routine of dairy farming and perceive it as satisfying.

Land organization is a debated issue in mountain regions due to the strong land fragmentation affecting these areas (39). It is possible that farmers satisfied with LO had more time to spend in animal care. Indeed, Fah is associated with certificated and loose-housed farms. Loose housing is perceived as more natural for the animals than tie-stall and, thus, increases the social acceptance of farming (63). On the other hand, quality scheme certification is also associated with social benefits (64, 65). Surprisingly, FA is little related to milk price (SL: 0.14). Mzoughi (66) highlights that not only financial, but also social compensation and recognition is essential for the satisfaction of the farmers. The above-cited authors explain that farmers try to reach personal satisfaction and recognition also by adopting and certifying ecologically friendly practices. High satisfaction related to both RNAC and RNC is positively linked to animal welfare, confirming that positive social engagement has an effect on animal care attitudes and practices.

## Conclusion

One Welfare is a recent theoretical approach aiming at mapping the interconnections between animal welfare, human well-being, and environmental health. A limited number of studies have explored the relationship between dairy cattle welfare and human well-being. To the authors knowledge, this is the first attempt to investigate the relationship between dairy cow welfare and farmer satisfaction in mountain areas. Despite the heterogeneity in the characteristics of small-scale mountain dairy farms involved in this study, the results show a generally good level of animal welfare. Animal welfare was higher in those farms where farmers have a positive engagement with both the agricultural and non-agricultural community and in those where farmers are satisfied with their land organization. The study contributes to shed light on the complex interconnections that exist among the different components of the One Welfare framework in the Eastern Alps. Further studies are needed at a larger scale to fully understand the links between animal and human well-being in mountain environments.

## Data Availability Statement

The original contributions presented in the study are included in the article/supplementary material, further inquiries can be directed to the corresponding author/s.

## Author Contributions

CS: data curation and writing-original draft. MB: data curation and writing –review-editing. MC: conceptualization and writing –review-editing. LG and MR: writing-review-editing. SP and WR: data curation. ES: writing-review-editing and funding acquisition. AZ: data curation, conceptualization, and writing –review-editing. SB: conceptualization, supervision, writing –review-editing, and funding acquisition. All authors contributed to the article and approved the submitted version.

## Funding

This work was supported by the Interreg V-A Italy-Austria 2014-2020 TOPValue (grant number ITAT2009).

## Conflict of Interest

The authors declare that the research was conducted in the absence of any commercial or financial relationships that could be construed as a potential conflict of interest.

## Publisher's Note

All claims expressed in this article are solely those of the authors and do not necessarily represent those of their affiliated organizations, or those of the publisher, the editors and the reviewers. Any product that may be evaluated in this article, or claim that may be made by its manufacturer, is not guaranteed or endorsed by the publisher.
